# Supplementary material for: The reach of commercially motivated junk news on Facebook
Source: PLoS One. 2019 Aug 1;14(8):e0220446. doi: 10.1371/journal.pone.0220446 (PMC6675076; doi:10.1371/journal.pone.0220446)
Supplement: S1 Appendix — (DOCX) [file pone.0220446.s001.docx]

# Appendix

**Table A: List of 20 Dutch mainstream news Facebook pages included in our sample**

| **URL** | **Name** | **Category** |
| --- | --- | --- |
| facebook.com/190231243842 | De Groene Amsterdammer | News magazine |
| facebook.com/103652819717294 | HP/De Tijd | News magazine |
| facebook.com/ad.nl | Algemeen Dagblad | National newspaper |
| facebook.com/bnr.nieuwsradio | BNR Nieuwsradio | News broadcast |
| facebook.com/decorrespondent | De Correspondent | Online news magazine |
| facebook.com/elsevierweekblad | Elsevier | News magazine |
| facebook.com/geenstijlnl | Geenstijl | Online news magazine |
| facebook.com/hetfd | Het Financieele Dagblad | National newspaper |
| facebook.com/metro | Metro | National newspaper |
| facebook.com/nos | NOS | News broadcast |
| facebook.com/nporadio1 | NPO Radio 1 | News broadcast |
| facebook.com/nrc | NRC | National newspaper |
| facebook.com/nu.nl | Nu.nl | Online newspaper |
| facebook.com/paroolnl | Het Parool | National newspaper |
| facebook.com/refdag | Reformatorisch Dagblad | National newspaper |
| facebook.com/rtlnieuws | RTL Nieuws | News broadcast |
| facebook.com/telegraaf | Telegraaf | National newspaper |
| facebook.com/tponl | The Post Online | Online newspaper |
| facebook.com/trouw.nl | Trouw | National newspaper |
| facebook.com/volkskrant | Volkskrant | National Newspaper |

**Table B: List of 63 Dutch junk news pages included in our sample**

| **URL** | **Name (if not in URL)** |
| --- | --- |
| facebook.com/106727739817828 | ROOS |
| facebook.com/1213051412048021 | Suri.nu |
| facebook.com/1315496065181384 | Ongelooflijk Favorieten |
| facebook.com/1364984373581269 | Originele Ideeën |
| facebook.com/1567673943543704 | Dierenvriend |
| facebook.com/1568382686520046 | Videodump |
| facebook.com/1661870274079781 | Kookfans |
| facebook.com/1721172708118846 | Viralfilmpje |
| facebook.com/1785829101687628 | LEESR |
| facebook.com/187487161609007 | Viraal.co |
| facebook.com/1931812020438753 | Dames&Heren |
| facebook.com/195089190827932 | Tips en Weetjes |
| facebook.com/386130775068830 | Dol op wilde dieren |
| facebook.com/410280812497912 | Doedatzelf |
| facebook.com/572385656268218 | Viralfy |
| facebook.com/662965343876026 | Ghettoland |
| facebook.com/688141944701187 |  |
| facebook.com/architectdistrict |  |
| facebook.com/arkinoh |  |
| facebook.com/bekijkdezevideo |  |
| facebook.com/bengbengnl |  |
| facebook.com/blijfpositiefcom |  |
| facebook.com/brawnl |  |
| facebook.com/breekdedag |  |
| facebook.com/curioctopus.nl |  |
| facebook.com/dagelijks.nu |  |
| facebook.com/deelze.nl |  |
| facebook.com/ditisgeniaal |  |
| facebook.com/echte.mannen.wereld |  |
| facebook.com/eetradar |  |
| facebook.com/feitjesenweetjes.nl |  |
| facebook.com/foodviral |  |
| facebook.com/forestfeed |  |
| facebook.com/gezondeideetjes |  |
| facebook.com/grappig.co |  |
| facebook.com/hetdelenwaard |  |
| facebook.com/indrukwekkend.nu |  |
| facebook.com/kingbreaknl |  |
| facebook.com/leeftips.nl |  |
| facebook.com/leeshetnu.nl |  |
| facebook.com/leeshetpuntnu |  |
| facebook.com/lhoriginale |  |
| facebook.com/lijpeshitt |  |
| facebook.com/livekijken |  |
| facebook.com/luidt.nl |  |
| facebook.com/newsnernederlands |  |
| facebook.com/nieuwsco |  |
| facebook.com/ongelofelijk.eu |  |
| facebook.com/pranksternl |  |
| facebook.com/secretmancave |  |
| facebook.com/straatvidsnl |  |
| facebook.com/toptrendingnl |  |
| facebook.com/trendnieuws |  |
| facebook.com/vandaagviraal |  |
| facebook.com/verhalen.co |  |
| facebook.com/viraaltjes |  |
| facebook.com/viraalvandaag |  |
| facebook.com/viral2day.nl |  |
| facebook.com/viralnextnieuws |  |
| facebook.com/viralsonline1 |  |
| facebook.com/viraltje |  |
| facebook.com/volgendevideo |  |
| facebook.com/zelfmaakideetjes |  |
